# T2-STIR CMR imaging can be used to assess myocardium at risk with gadolinium present in an experimental setting

**DOI:** 10.1186/1532-429X-15-S1-P208

**Published:** 2013-01-30

**Authors:** David Nordlund, Mikael Kanski, Robert Jablonowski, Joey F Ubachs, Sasha Koul, Jesper van der Pals, Marcus Carlsson, David Erlinge, Hakan Arheden, Henrik Engblom

**Affiliations:** 1 Clinical physiology, Skane University Hospital, Lund University, Lund, Sweden; 2 Cardiology, Skane University Hospital, Lund University, Lund, Sweden

## Background

In the situation of an acute coronary occlusion, the myocardium supplied by the affected artery becomes ischemic. This part of the myocardium is at risk of developing infarction unless reperfusion occurs, and is therefore referred to as the myocardium at risk (MaR). Myocardium at risk and infarct size can be used to calculate myocardial salvage when evaluating efficacy of interventional or cardioprotective therapies. Cardiovascular magnetic resonance (CMR) has been shown to enable quantification of MaR using T2-weighted CMR and has been validated for use in patients. There are few studies validating T2-weighted CMR for ex vivo quantification of MaR in experimental studies, especially with presence of a gadolinium-based contrast agent needed for assessment of infarct size. The purpose of this study was to test if the clinically validated T2-weighted short tau inversion recovery (T2-STIR) imaging sequence can be used to assess MaR with gadolinium present in an experimental ex vivo setting.

## Methods

Acute ischemia was induced in 26 pigs for 40 minutes followed by 4 hours of reperfusion using a closed-chest, catheter-based experimental model. After explantation and preparation of the heart, T2-STIR CMR images were acquired for quantification of MaR. As reference standard for MaR, ex vivo myocardial perfusion SPECT was used. For assessment of infarct size, high resolution T1-weighted CMR images were acquired.

## Results

**Figure 1:**
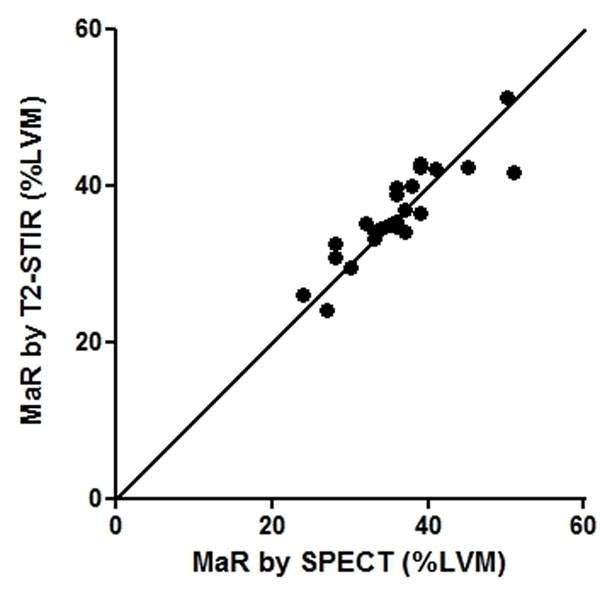


A strong correlation between MaR by T2-STIR CMR imaging and myocardial perfusion SPECT was found (r2=0.81, p<0.0001) and no significant difference was seen (p: 0.1013). In addition, a strong correlation was found between a central core zone of the MaR and infarct size as assessed by T1-weighted CMR (r2=0.82, p<0.0001, Figure 1).

## Conclusions

T2-STIR can be used to quantify MaR in an experimental ex-vivo setting with gadolinium present. Furthermore, quantification of the MaR core zone on T2-STIR may enable quantification of infarct size and, consequently, myocardial salvage.

## Funding

Swedish research council, swedish heart lung foundation, region of skane, medical faculty lund university

